# Supplementary material for: A Novel Core Effector Vp1 Promotes Fungal Colonization and Virulence of Ustilago maydis
Source: J Fungi (Basel). 2021 Jul 23;7(8):589. doi: 10.3390/jof7080589 (PMC8396986; doi:10.3390/jof7080589)
Supplement: Supplementary file 1 [file jof-07-00589-s001.zip › jof-1316640-supplementary.pdf]

A novel core effector Vp1 promotes fungal colonization and virulence of *Ustilago maydis*

Cuong Van Hoang[1,2,3], Chibbhi K. Bhaskar[1,2,3], Lay-Sun Ma[1,2,4*]

[1] Institute of Plant and Microbial Biology, Academia Sinica, Taipei 11529, Taiwan;
[2] Molecular and Biological Agricultural Sciences Program, Taiwan International Graduate Program, National Chung Hsing University and Academia Sinica, Taipei 11529, Taiwan;
[3] Graduate Institute of Biotechnology, National Chung-Hsing University, Taichung 402, Taiwan;
[4] Biotechnology Center, National Chung Hsing University, Taichung 402, Taiwan;
***** Correspondence: laysunma@gate.sinica.edu.tw; Tel.: +886-2-2787-1145

Supporting information

Figure S1- UmVp1 is important for *U. maydis* virulence
Figure S2- UmVp1 is not glycosylated
Figure S3- The analysis of Vp1 orthologs in smut-fungi
Figure S4- Secretion of Vp1-mCherry protein in plant apoplastic space
Figure S5- Subcellular localization of the GFP fused Vp1 proteins in maize protoplasts from healthy plants
Figure S6- Subcellular localization of GFP fused UmVp1, UhVp1 and UmVp1-nls proteins in *N. benthamiana* leaves
Figure S7- Gene expression profiling of sr*vp1* by qRT-PCR

Table S1- Primers used in this study
Table S2- *U. maydis* strains used in this study

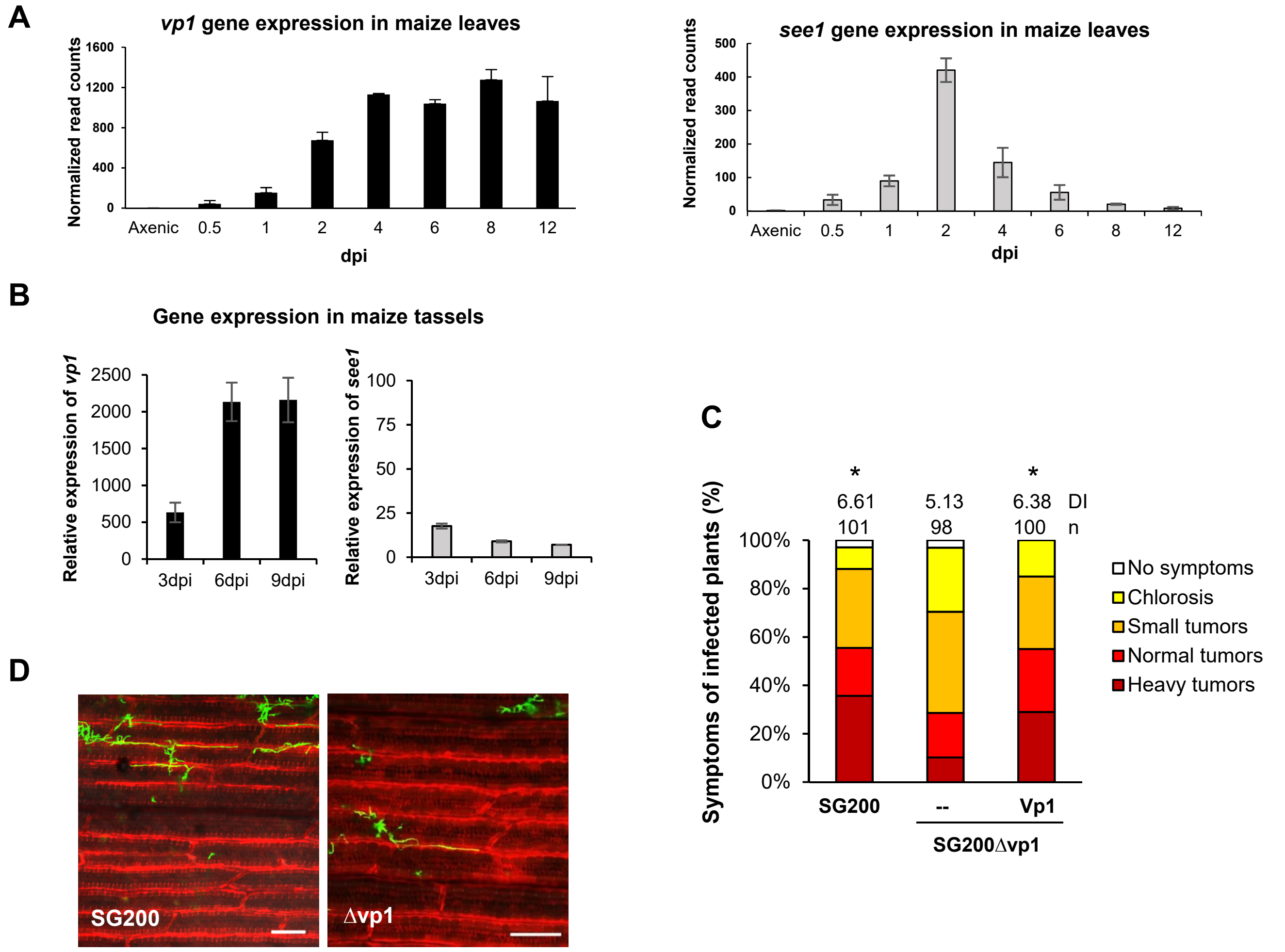

Figure S1. UmVp1 is important for *Ustilago maydis* virulence. The expression patterns of *umvp1* (*UMAG00538*) and *see1* (*UMAG02239*) genes were retrieved from RNA sequencing data generated from the maize leaves infected by the compatible FB1xFB2 strains [Lanver et al., 2018]. Error bars indicate ±SD. **B.** Gene expression profiling of *vp1* and *see1* in maize tassels by qRT-PCR. The expression of *see1* gene not detected in tassels was served as negative control [Redkar et al., 2015]. Total RNA was extracted from maize tassels infected with SG200 at 3, 6 and 9 dpi. The constitutively expressed *U. maydis* peptidylprolyl isomerase (*ppi*) gene was used for normalization. The data represent the mean obtained from three independent experiments and the error bars depict ± SD. **C.** The 7-day-old-maize seedlings of EGB variety were infected with the indicated strains and disease symptoms were scored at 10 dpi. n, total numbers of infected plants from the three independent infection assays; DI, average disease index of three independent infection assays; *P<0.05 indicates significant differences of disease symptoms in respective complementation strains compared with Δumvp1 determined by a two-tailed Student's *t-test* using the disease index values. **D**. Maize seedlings infected by the indicated *U. maydis* strains were observed at 2 dpi by confocal microscopy. Fungal hyphae were stained with WGA-AF488 (green). Plant cell walls were stained with propidium iodide (red). Bars: 200 μm.

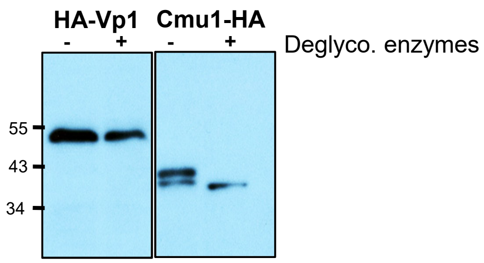

Figure S2. UmVp1 is not glycosylated. The TCA-precipitated HA-Vp1 or Cmu1-HA proteins were treated with (+) or without (-) deglycosylation enzyme mix (containing PNGase F, *O*-glycosidase, β1-4 galactosidase, β-N-acetylglucosaminidase, and neuraminidase) according to the manufacturer´s protocol (NEB Cat# P6039). Cmu1-HA served as positive controls [Djamei et al., 2011]. The protein samples were separated on SDS-PAGE and analyzed by western blotting using the anti-HA antibody.

Figure S3

A

```
                 *        20         *        40         *        60         *        80         *
Tc  : -MRRHHRHSLSCVTRRFGLVLLLFLLVAWISAYDLD-KDGTASIGAYYVQPGRCAGVEKTLRW-RYRYFFRPNIPETVYP--------------- :  77
Ub  : MIA----------RFT-NLILVVIGIATGVLSYKLSPDDGQADIGAYHVQPGRCASVERRLGRTKYDYFFHSDPTITSFTPGTPSNPSPP----- :  79
Uh  : MIA----------RFT-NLILVVIGIATGVLSYKLSSDDGQADIGAYHVQPGRCASVERRLGRTKYDYFFHSDPTITSFTPGTPSNPSPP----- :  79
Ut  : -MS----------RLSSVLSLFVILSSSRVMSYMLSPIDGQADIGAYHVQPGRCADVEKHLGRKVYEKFFLSSPFQTSFTPLGPESYDPNPPRNP :  84
Map : ------MLS---MKLFVVPATVVLACLCKAAAYKLDAEDGQAEIGAYHIQPGRCAGVEQRLGHTKYSQYYHWSPFHTSFTELG------------ :  74
Man : ------------MKLFVVPVTVVLACLCNAAAYKHDAEDGQAEIGAYHIQPGRCAGVEQRLGHTKYSQYYHWSPFHTSFTELG------------ :  71
Mp  : MM---TVAP---FYLSLRLWLVLLTASSTVLTYELSPVDGQADIGAHHVQPGRCAGVERRLGRRTYGDFFRPSPFKTTFSSISS---S------- :  79
Sr  : MG---ERSG----RLLLYITVLVMLVSPRVLGYKLSPVDGQADIGAYHVQPGRCAGVESRLRT-KYRYFFGASPFHTTFDQIVHPDPT------- :  80
Ss  : MT---LRPQ---LTSLTLCVVLISLLSLHITAYKLSPVDGQADIGAYHVQPGRCAGVEQNLRR-KYRYFFG-SPFHTTFDQIHPT---------- :  77
Um  : MLARLWQSS---VTSLVPLLLRLILAPSYITAYHLSPTDGQADIGAYHVQPGRCAGVERRLGKARYSYFFGPSPFQTTFS----ADSS------- :  81
Ph  : MITRSVQST---FALLLVTVTLLIASSPPVASYHLSPDDGQADIGAYHVQPGRCADVERRLGR-RYSYFWRSSPFFTTFT----PDPS------- :  80
                            6         Y l   DGqA IGAyh6QPGRCA VE  L    Y  55   p  T 5

        100         *       120         *       140         *       160         *       180         *
Tc  : --------GKLDINDMDSTFLCSWDPQLTEGYGGSFDGENPYLLYARCHRGTNEVVALCDTIDDKGKPSKIV-SPV-KATCPAGTRCKNFCATMQ : 162
Ub  : -SPPSNGEAGSGIEQMDATALCSFNPELTQGYGGGFDEMKLYLVYSRCLYGTETMRVLCGTTNAHGKPIAS-YNVV-QATCPAGTRCKNFCATMQ : 171
Uh  : -PPPSNGEAGSGIEQMDATALCSFNPELTQGYGGGFDEMKLYLVYSRCLYGTETMRVLCGTTNAHGKPIAS-YNVV-QATCPAGTKCKNFCATMQ : 171
Ut  : GTHPDVNGAGSGIEAMDATSLCSFNPELTEGYGGGFDKMKLYLVYSRCIYGTEQMRVLCGSTDGKGRPDKI-VNIV-QAGCPTGTKCKNICATMR : 177
Map : --------YGNDVEQMDSTALCANNPALTEGYGGGFDKMRIYLVYGRCVYGTQDMKVMCGTITEQGRPSHNILNVV-QASCPVGTRCKNICATMQ : 160
Man : --------YGNDVEQMDSTALCANNPALTQGYGGGFDKMRIYLVYGRCVYGTQDMKVMCGTITEQGRPSHNILNVV-QASCPAGTRCKNICATMQ : 157
Mp  : --ISGVDDAGSGIEAMDSTSLCSFNPRLTEGYGAGFSNIASYLIYSRCLYGTNYMRVICGTSDGNGKPKEEIPNIVVEAQCPSGTKCKNFCATMV : 172
Sr  : --VSSSAGSGTNVEQMDATALCAFNPLLTEGYGGGFDSLNFYLVYSRCIYGTDEMRVLCGTTDGKGVPIDSYFNLI-QAKCPQGTRCKNICASMT : 172
Ss  : ----------SDSDQLDATALCSFNPLLTEGYGGGFDNINFYLVYSRCIYGTDDMRVLCGTTDGKGVPINSYYNLI-QARCPRGTKCKNICASMV : 161
Um  : --SS-SQDAGLGIEQMDSTSLCAFDPELTEGYGAGFDNMRFYLVYSRCIYGTNEMRALCGTTDGKGKPIDKIFNLV-QATCPRGTRCKNICATMH : 172
Ph  : --SSLIPTAGSGVEQMDATSLCTSDPLINEGYGAGFDKMRLYLVYSRCLFGTNEMRVLCGTIDDKGKPMNSYFNLV-QAGCPQGTRCKNICATMR : 172
               g   e 6D T LC  1P 6t2GYG gFd    YL6Y RC  GT  6 v6Cg3    G P     n 6  A CP GT4CKN CA3M

             200         *       220         *       240         *       260         *       280
Tc  : DPSKANRYAAQQVQLTQCLPDAEWSELTDKYRPKSVANAAQAPDQ-----QQPAGPAKP-----------LPANPGT--ASGGGDGTVTRLSVGK : 239
Ub  : DPSLTSPFAAKQVQLAQCLPIDQWQKLTDMYKPKSPPSLSNAAPGG-------GGNRADAKVDPKVVEKVPEGDPTKDPTHVDKDGTIAGKVLSP : 259
Uh  : NPSLTSPFAAKQVQLAQCLPIDQWQKLTDMYKPKSPPSLSNAAPGG-------GGNRADAKVDPKVVEKVPEGDPTKDPTHVDKDGTIAGKVLFP : 259
Ut  : DPSLTSQYAAPQVQLAQCMELDYWQKLTDMYKPKVPVQPGSPTSKA-------EGDR----SEPKVVDKAPK-EPTTDQMRPDQDGTIPGKVINP : 260
Map : DPSLTSKFAVKQVQLAQCMPIPQWNKLTNMYRPKASPLPNAN----------QAATAASPKIDPKVVDKVPTGNPTVDPVHVDNDGTIPGRVVSP : 245
Man : DPSLTSKFAVKQVQLAQCMPIPQWKKLTEMYRPKASPVPAAY----------QVAAAGSQKIDPKVVDKAPAGNPTVDPVHVDKDGTIPGRVVSP : 242
Mp  : DPSLASPYAAKQVQLAQCLPIPQWEKLTDMYKPKPRWFNYDSLLK-----IKTGGGVGSAGGDPKVVDKVPEGDPAKDPTQTDKDGTIAGKVLIS : 262
Sr  : DPKLTSPYAAKQVQLAQCMPDAQWQHLTDMYRPKTDSGPAG-----GKQPEKPE-ATNVPKVDPTVVDKPP-ANPTKDETKPAKDGTIAGKVLPA : 260
Ss  : DPKLTSPYAAKQVQLAQCMPETQWQTLTDMYRPKNPTEKEA-----KGGAAKPE-AAKVPKIDPTVVDKTPEKDPTKDQTQTDGDGTIAGKVLPK : 250
Um  : DPTLTSPYAAKQVQLAQCMSEQQWQFLTDMYRPKSALTKPAA-QMSAPQAANPHSAQTDGRLDPTVVDKVPASNPHHDQTHTDQDGTIAGKVLPL : 266
Ph  : DPKLTSPYAAKQVQLAQCMDEKQWQKLTDMYRPKPATLAVT-------------VAPPPSHLDPQVVDKVPSKNPTQDQTKPDQDGTIAGQVLSP : 254
      1P lts 5AakQVQLaQC6    W  LT mY4PK                            dp vv k p   P  d    d DGT6 g v6

          *       300         *       320         *       340         *       360         *       380
Tc  : GTVGVSLVGNEITSPDHAHL--------NPSNPTPQTGTAE---THTH--------------------------------------HDPSSDNDF : 285
Ub  : APASPKTDDKAPPSPPSKKEEQE-------RAPSPQPDAQQGEKPITVGHLQVGAGSAPKA---------------DPKKPEPPAAANPKGD--F : 330
Uh  : AHASPKTDDKAPPSPPSKKEEQE-------PAPSPQRDAQQGKKPITVGHLQVGAGSAPKP---------------DPKKPEPPAAANPKGD--F : 330
Ut  : GTGANQPSN--PPEDPNTQHGPKASTPPKPEAASPNSVPQPQNPPHTTGHLVFAGGFSAFSLYGQP----------QPAQPEPQPA-QPEPE--P : 340
Map : STPSTSDIDAALLKA------------------------ANDKEAYSGERL-----VTKQPD--GQKP-V-AAQPTKGD-SDLPTG--------- : 297
Man : STPPTDDIDADLLKA------------------------ANEREAYSGERL-----VTKQPD--VEKP-V-PAQPAKGDSSDLPIG--------- : 295
Mp  : GASDAKPESKKQPK--------------------S----KTDTGMHTTTHLSEGLTFP---GSVH-----------------M------------ : 301
Sr  : PADAKKDDAAKPAEPPKQPA------P----APAPAT--GKDDAAHTTGHFQISPIVILMPQPQLNPPKEGDKPPAQPAKAEPPKAA-------- : 335
Ss  : ADGDKGKA-DKTPPPAQAPA------PAPAPAPAPDKGNTGQPEPHTTGRFQTSPFVILLPQIEQ--PPKDDQKPAQPAKPAPPTAADPKAPKPA : 336
Um  : AAA------------------------------------GRPAGPYTTGHLQLPPNAK-------------PNTPKQAVDSSPPSS--------- : 303
Ph  : TAA------------------------------------GQSEPGHTTGHLQLDPKQP-------------PAPPTQNDQPKADQ---------- : 290
                                                    3

               *       400         *       420         *       440         *       460         *
Tc  : DN----------IVT------ITTVKASNPKSQPGLSRMGF------------------------------------------------------ : 310
Ub  : GPLAAGHGPTNPILL---LEPSGQNPQRPLERQKAPTEIGF------------------------------------------------------ : 368
Uh  : GPLAAGHGPTNPILP---LEPSGQNPQRLLERQKAPTEMGF------------------------------------------------------ : 368
Ut  : EP-----EP----------KQPEQAQPDKLPPQKAPTDMGFRKRHLQKDGSLSLS----------------HHRHRHGRSARL------------ : 392
Map : -----GPQFVPAAANAPVE---PQTNGHKLEHQQAPTRMGFRKRSLSGALNS------------------------------------------- : 341
Man : -----GPQFVPAAKA--PE---AQTNGHKLEHQQAPTRMGFRKRSLSGALNA------------------------------------------- : 337
Mp  : --------PTGNKPNDPIEQQQKQDQKDKLPPQKALTDMGFRKRTLQYDLSSSSGSGTRGFIHSFHTGQMKHDPHKGQQSKRSADPASQITIPLS : 388
Sr  : DPKAADPKPADPKAAGPDAG----KDDGKLPPQKAPTAMGFRKRSLS--SGFRHH-----------------RRSAAAV---------------- : 391
Ss  : QPKAADPKPADPKATDPKDT--SADKQSKPPPQKAPTPMGFRKRSLR--EGSRRH-----------------RRSASAHV--------------- : 395
Um  : ------------PAQSPAEPD-APGDEATRQTQKAPTPMGFRKRSVPESSGELRR-----------------HRSKTVAAR-------------- : 354
Ph  : -------------------GA-DSSKDIKDPPQKAPTVMGFRRRSLDFSVPSRRH-----------------DPHGHRSSRAPVDARADS----- : 343
                                      Q ap3 6GF

        480         *       500         *       520
Tc  : ---------------------------------------------------- :   -
Ub  : ---------------------------------------------------- :   -
Uh  : ---------------------------------------------------- :   -
Ut  : ---------------------------------------------------- :   -
Map : ---------------------------------------------------- :   -
Man : ---------------------------------------------------- :   -
Mp  : SVDKPIQPKAQQQQHEKDPDGPLTPQKDPLPGKKGPLHRHHRHLRSRILHQH : 440
Sr  : ---------------------------------------------------- :   -
Ss  : ---------------------------------------------------- :   -
Um  : ---------------------------------------------------- :   -
Ph  : ---------------------------------------------------- :   -
```

Figure S3

B

Orthologs	Coverage (%)	Identity (%)	E value for RBH
Pseudozyma hubeiensis XP_012192856.1	80	58.8	6e-121
Sporisorium reilianum CBQ67951.1	94	53.3	2e-109
Sporisorium scitamineum CDU24160.1	93	50.77	3e-105
Melanopsichium pennsylvanicum CDI51462.1	75	50.14	1e-93
Moesziomyces antarcticus XP_014659418.1	92	45.87	4e-86
Moesziomyces aphidis ETS63783.1	91	47.87	7e-88
Ustilago hordei CAJ41947.1	64	59.35	4e-93
Ustilago trichophora SPO21134.1	67	56.47	6e-91
Ustilago bromivora SAM61624.1	66	60.2	8e-96
Testicularia cyperi PWY98796.1	65	52.34	2e-57

C

	Vp1 orthologs	Signal peptide	Probability
1	*Ustilago maydis*	MLARLWQSSVTSLVPLLLRLLLAPSYITA	0.6638
2	*Pseudozyma hubeiensis*	MITRSVQSTFALLLVTVTLLLASSPPVAS	0.8060
3	*Sporisorium reilianum SRZ2*	MGERSGRLLLYITVLVMLVSPRVLG	0.9728
4	*Sporisorium scitamineum*	MTLRPQLTSLTLCVVLLSLLSLHITA	0.8533
5	*Ustilago bromivora*	MIARFTNLILVVIGIATGVLS	0.8605
6	*Ustilago hordei*	MIARFTNLILVVIGIATGVLS	0.8044
7	*Melanopsichium pennsylvanicum 4*	MMTVAPFYLSLRLWLVLLTASSTVLT	0.7058
8	*Moesziomyces aphidis*	MLSMKLFVVPATVVLACLCKAAA	0.7541
9	*Ustilago trichophora*	MSRLSSVLSLFVLLSSSRVMS	0.9201
10	*Moesziomyces antarcticus T-34*	MKLFVVPVTVVLACLCNAAA	0.6159
11	*Testicularia cyperi*	MRRHHRHSLSCVTRRFGLVLLLFLLVAW ISA	0.8794

Figure S3. The analysis of Vp1 orthologs in smut-fungi. **A**. The amino acid sequence alignments of Vp1 orthologs in related smut fungi. The full length sequences of the orthologs were obtained from NCBI database, aligned using Clustal Omega, and visualized using Genedoc. The predicted N-terminal signal peptides are boxed in green. The orange line indicates the predicted NLS in each ortholog by LOCALIZER (http://localizer.csiro.au/). The red line indicates the conserved N-terminal regions among othologs. The blue dashed line indicates the disorder region of UmVp1 predicted by D^2P^2 (https://d2p2.pro/). *Testicularia cyperi* (Tc) [Kijpornyongpan et al., 2018] ; *U. bromivora* (Ub) [Rabe et al., 2016]; *U. hordei* (Uh) [Laurie et al., 2012]; *U. trichophora* (Ut) [Zambanini et al., 2016]; *Moesziomyces aphidis* (Map) [Lorenz et al., 2014]; *Moesziomyces antarcticus* (Man) [Morita et al., 2013]; *Melanopsichium pennsylvanicum* (Mp) [Sharma et al., 2014]; *Sporisorium reilianum* (Sr) [Schirawski et al., 2010]; *Sporisorium scitamineum* (Ss) [Dutheil et al., 2016]; *Ustilago maydis* (Um) [Kamper et al., 2006]; *Pseudozyma hubeiensis* (Ph) [Konishi et al., 2013]. **B**. The Vp1 orthologs identified in smut fungi through a blastP analysis and reciprocal best blast hits (RBH) and OrthoDB search. **C**. Signal peptide sequences of Vp1 orthologs predicted by SignalP5.0 (http://www.cbs.dtu.dk/services/SignalP/) are shown.

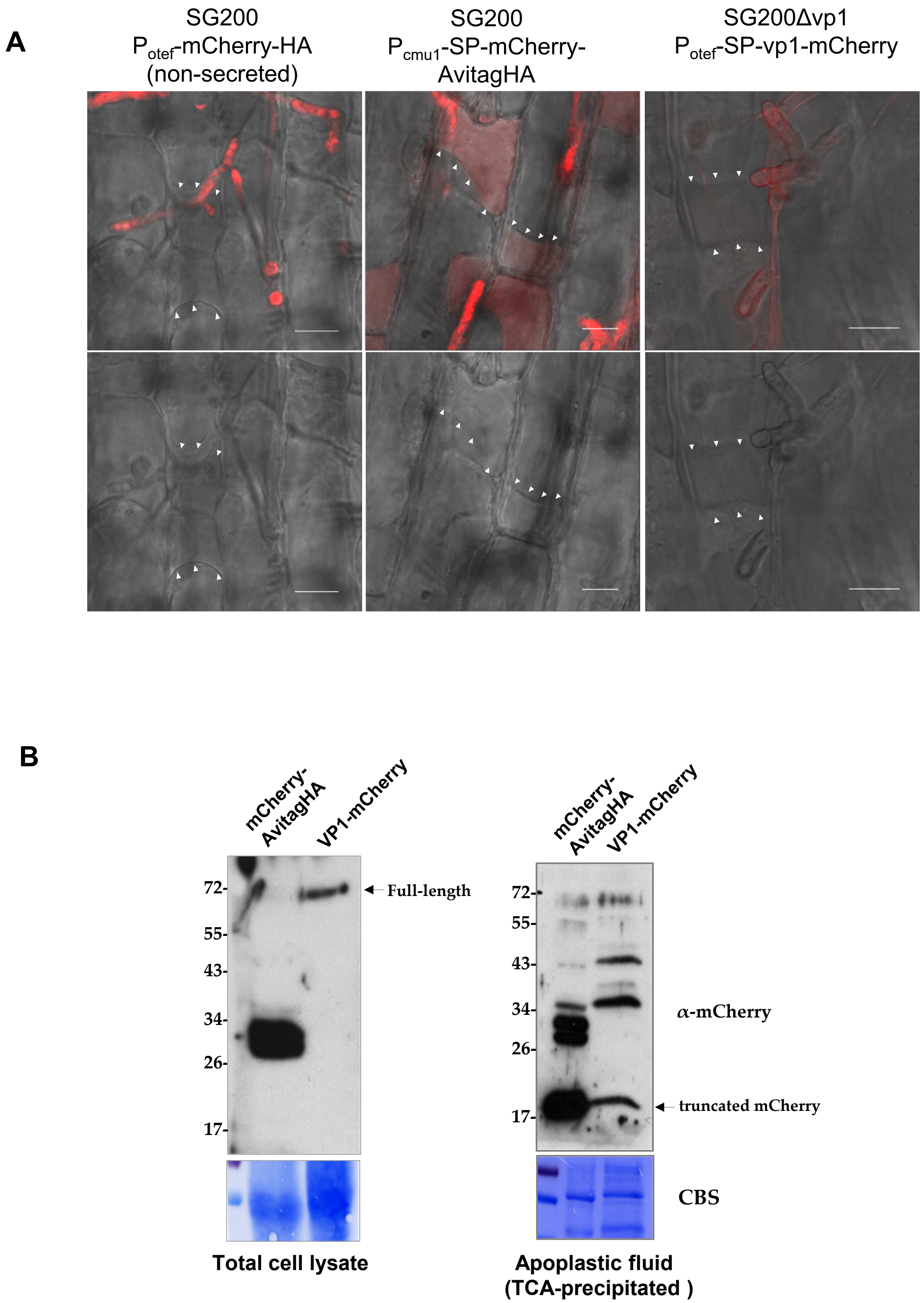

Figure S4. Secretion of Vp1-mCherry protein in plant apoplastic space. **A.** Maize leaves infected with the indicated strains were collected at 3 dpi. The mCherry fluorescence signals in plasmolysis area (indicated by arrows) were visualized by confocal microscopy (excitation at 561 nm and detection at 580-630 nm) after the leaves treated with 1M NaCl. Bar, 10 µm. **B**. Infected leaves were collected at 3 dpi and the total proteins extracted by sample buffer and analyzed in immunoblots using antibody against mCherry epitope (Total cell lysate). Total proteins from apoplastic fluid were collected from infected leaves using vacuum-centrifugation method and TCA-precipitated. CBS: Coomassie blue staining as a loading control.

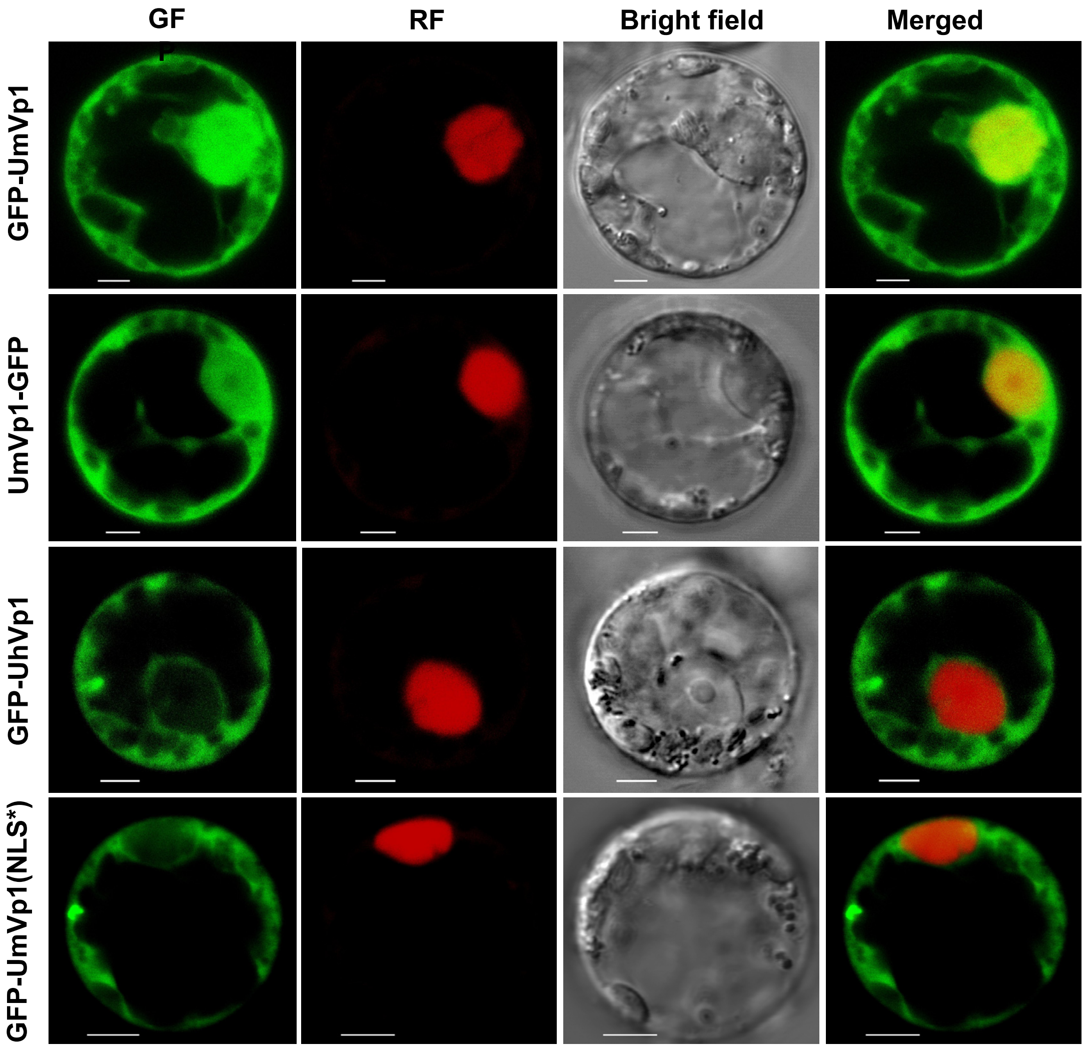

Figure S5. Subcellular localization of the GFP fused Vp1 proteins in maize protoplasts from healthy plants. GFP-tagged Vp1 and the nuclear marker 35s-RFP-VirD2NLS were co-expressed in maize protoplasts and visualized by confocal microscopy. Bars, 5 μm.

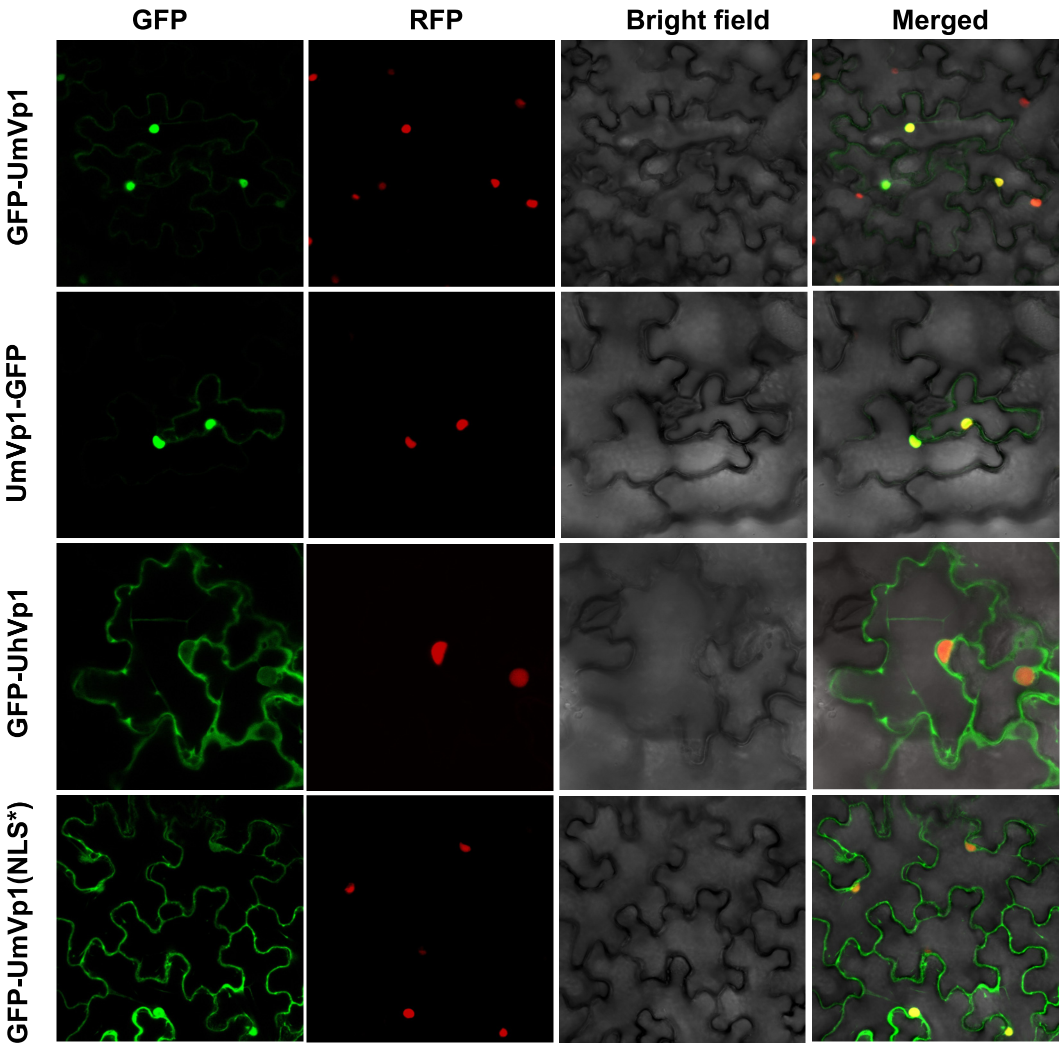

Figure S6. Subcellular localization of GFP fused UmVp1, UhVp1 and UmVp1-nls proteins in *N. benthamiana* leaves. The GFP-tagged Vp1 and the nuclear marker 35s-RFP-VirD2NLS were co-expressed *N. benthamiana* leaves and observed after 2 days of inoculation and visualized by confocal microscopy.

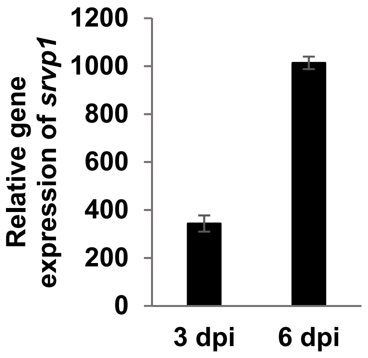

Figure S7. Gene expression profiling of sr*vp1* by qRT-PCR. Total RNA was extracted from leaves infected with SG200Δvp1-srvp1 at 3 and 6 dpi. The constitutively expressed *U. maydis* peptidylprolyl isomerase (*ppi*) gene was used for normalization. The data represent the mean obtained from three independent experiments and the error bars depict mean ± SD.

Table S1. Primers used in this study

Plasmids	Primers	Sequences (5'-3')
**Generating vp1 mutant**	#1	GCTCGAGTTTTTCAGCAAGATAATATTGTTCAGGACAAAGCGGTATCTC
pJET-vp1KO	#2	CGCAATTGTCACGCCATGGTGGCCATCTAGGCCTCCGAGAGCTTGCCGCTCGTAG
	#3	CTGTGCGGCCGCATTAATAGGCCTGAGTGGCCAAGCTCCAGAAAGCTTGAC
	#4	AGGAGATCTTCTAGAAAGATAATATTGTGCCGAGCCACTGATATG
**Complementation**	#5	GCCATATGTTGTTCAGGACAAAGCGGTATCTC
p123-vp1	#6	ATGCGGCCGCTTTAACGAGCAGCGACTGTTTTG
p123-HA-vp1	#7	GCTTAACTATGCGGCAT
	#8	ATAGTCGGGGACGTCGTAGGGATACGCCGTAATGTACGATGG
	#9	CCTACGACGTCCCCGACTATGCCTACCACCTGTCACCTACTG
	#10	AAGACCGGCAACAGGATTCAATC
p123-otef-HA-vp1	#11	TCCCCCGGGATGTTGGCACGACTCTGG
	#6	ATGCGGCCGCTTTAACGAGCAGCGACTGTTTTG
p123-gfp-vp1	#7	GCTTAACTATGCGGCAT
	#12	CCTTGCTCACCGCCGTAATGTACGATGGTG
	#13	CATTACGGCGGTGAGCAAGGGCGAGGAGCTG
	#14	GGTATCTAGACTTGTACAGCTCGTCCATGC
	#15	GCTGTACAAGTCTAGATACCACCTGTCACCTACTG
	#16	AAGACCGGCAACAGGATTCAATC
p123-vp1-gfp	#7	GCTTAACTATGCGGCAT
	#17	GCCCTTGCTCACTCTAGAACGAGCAGCGACTGTTTTGC
	#18	GTTCTAGAGTGAGCAAGGGCGAGGAG
	#19	CGATCTGCAGCCGGGCGGCCGCTTTACTTGTACAGCTCGTCC
p123-Uhvp1	#7	GCTTAACTATGCGGCAT
	#20	CGAGAGCTTGCCGCTCGTAG
	#21	CAAGCTCTCGGAATGATTGCTCGATTCACCAATC
	#22	CGATCTGCAGCCGGGCGGCCGCTCTAGAAGCCCATCTCCGTG
p123-Srvp1	#7	GCTTAACTATGCGGCAT
	#20	CGAGAGCTTGCCGCTCGTAG
	#23	CAAGCTCTCGGAATGGGTGAGCGATCCGGGCG
	#24	CGATCTGCAGCCGGGCGGCCGCTTCAAACGGCAGCAGCGCTGC
p123-Ssvp1	#7	GCTTAACTATGCGGCAT
	#20	CGAGAGCTTGCCGCTCGTAG
	#25	CTACGAGCGGCAAGCTCTCGGAATGACTTTGCGACCCCAGCTG
	#26	GATCTGCAGCCGGGCGGCCGCTTCACACGTGCGCAGAAGCAC
p123-Mpvp1	#7	GCTTAACTATGCGGCAT
	#20	CGAGAGCTTGCCGCTCGTAG
	#27	CTACGAGCGGCAAGCTCTCGGAATGATGACGGTGGCTCCTTTC
	#28	GATCTGCAGCCGGGCGGCCGCTTCAATGCTGGTGGAGGATAC
p123-SP_{vp1}-Srvp1	#7	GCTTAACTATGCGGCAT
	#29	GACAGCTTGTACGCCGTAATGTACGATGGTG
	#30	CATTACGGCGTACAAGCTGTCTCCCGTGG
	#24	CGATCTGCAGCCGGGCGGCCGCTTCAAACGGCAGCAGCGCTGC

p123-SP$_{vp1}$-Uhvp1	#7	GCTTAACTATGCGGCAT
	#31	GACAGCTTGTACGCCGTAATGTACGATGGTG
	#32	CATTACGGCGTACAAGCTGTCGTCAGACG
	#22	CGATCTGCAGCCGGGCGGCCGCTCTAGAAGCCCATCTCCGTG
p123-vp1-nls*	#7	GCTTAACTATGCGGCAT
	#33	GAAAGCCGGAAGAAAAAGGCACACTAGCCGCGGCGAAGCCCATCGGTGTC
	#34	GCCTTTTTCTTCCGGCTTTCTTGCTGCCCACGCAAGCGCAACAGTCGCTGCTCG
	#35	CCTTTGAGTGAGCTGATACC
p123-otef-dSP-HA-vp1	#45	TCCCCCGGGATGTATCCCTACGACGTCCCC
	#6	ATGCGGCCGCTTTAACGAGCAGCGACTGTTTTG
p123-vp1(Δnls)	#5	GCCATATGTTGTTCAGGACAAAGCGGTATCTC
	#46	GGGCGGCCGCCTAGAAGCCCATCGGTGTCGGTG
p123-otef-vp1-HA	#11	TCCCCCGGGATGTTGGCACGACTCTGG
	#47	CGGGGACGTCGTAGGGATATCTAGAACGAGCAGCGACTGTTTTG
	#48	GATATCCCTACGACGTCCCCGACTATGCCTAGGCGGCCGCCCGGCTG
	#35	CCTTTGAGTGAGCTGATACC
p123-otef-vp1(Δnls)-HA	#11	TCCCCCGGGATGTTGGCACGACTCTGG
	#49	GACGTCGTAGGGATATCTAGAGAAGCCCATCGGTGTCGGTG
p123-otef-vp1-mCherryHA	#50	GTTCTAGAAAGGGCGAGGAGGATAACATG
	#51	GGCGGCCGCTTAAGCGTAATCTGGAACATC
p123--otef-vp1(nls*)-mCherryHA	#11	TCCCCCGGGATGTTGGCACGACTCTGG
	#52	CTTTCTAGAACGAGCAGCGACTGTTGC
p123-otef-vp1-mCherry	#50	GTTCTAGAAAGGGCGAGGAGGATAACATG
	#53	CCGCGGCCGCTTTACTTGTACAGCTCGTCCATG
**Localization**	#36	CAAGCTAATTCTGCAGTCGACATGGTGAGCAAGGGCGAGGAG
pEZRK-gfp-vp1	#37	CATTAAAGCAGGACTCTAGATTAACGAGCAGCGACTGTTTTG
pEZRK-vp1-gfp	#38	GAGCTCAAGCTAATTCTGCAGTCGACATGTACCACCTGTCACC
	#39	CATTAAAGCAGGACTCTAGATTACTTGTACAGCTCGTCCATG
pEZRK-gfp-Uhvp1	#40	CATGGACGAGCTGTACAAGTCTAGATACAAGCTGTCGTCAGAC
	#41	CTCATTAAAGCAGGACTCTAGACTAGAAGCCCATCTCCGTG
pEZRK-gfp-vp1(nls*)	#36	CAAGCTAATTCTGCAGTCGACATGGTGAGCAAGGGCGAGGAG
	#42	CATTAAAGCAGGACTCTAGATTAACGAGCAGCGACTGTTG
pEZRK-rfp-virD2nls	#43	GCTCAAGCTAATTCTGCAGTCGACATGGCCTCCTCCGAGGAC
	#44	CTCATTAAAGCAGGACTCTAGATTATAGGTGGATCCCGACGC
**Gene expression and fungal biomass**	ppi-F	ACATCGTCAAGGCTATCG
	ppi-R	AAAGAACACCGGACTTGG
	GAPDH-F	CTTCGGCATTGTTGAGGGTTTG
	GAPDH-R	TCCTTGGCTGAGGGTCCGTC
	vp1-F	GGTCATCTACAACTGCCTCCCA
	vp1-R	CGGTGCTTTCTGTGTCTGTC
	See1-F	TCAGGTGCAAGGAGAAGG
	See1-R	ACAGAATACTCCGCTTCCC
	Srvp1-F	CACCGGGCACTTTCAAATATC
	Srvp1-R	CTTTTGGTGGTTCCGCTTTCG

Table S2. *U. maydis* strains used in this study

Strain name	Genotype	Reference
SG200	*a1 mfa2 bW2*	Müller et al., 1999
SG200Δvp1	*a1 mfa2 bW2 Δumag00538 (vp1)*	This study
SG200Δvp1-vp1	*a1 mfa2 bW2 Δvp1* $ip^r[P_{vp1}:vp1]ip^s$	This study
SG200Δvp1-HA-vp1	*a1 mfa2 bW2 Δvp1* $ip^r[P_{vp1}:HA\text{-}vp1]ip^s$	This study
SG200Δvp1-otef-HA-vp1	*a1 mfa2 bW2 Δvp1* $ip^r[P_{otef}:HA\text{-}vp1]ip^s$	This study
SG200Δvp1-gfp-vp1	*a1 mfa2 bW2 Δvp1* $ip^r[P_{vp1}:gfp\text{-}vp1]ip^s$	This study
SG200Δvp1-vp1-gfp	*a1 mfa2 bW2 Δvp1* $ip^r[P_{vp1}:vp1\text{-}gfp]ip^s$	This study
SG200Δvp1-vp1(nls*)	*a1 mfa2 bW2 Δvp1* $ip^r[P_{vp1}:vp1nls^*]ip^s$	This study
SG200Δvp1-uhvp1	*a1 mfa2 bW2 Δvp1* $ip^r[P_{vp1}:uhvp1]ip^s$	This study
SG200Δvp1-srvp1	*a1 mfa2 bW2 Δvp1* $ip^r[P_{vp1}:srvp1]ip^s$	This study
SG200Δvp1-ssvp1	*a1 mfa2 bW2 Δvp1* $ip^r[P_{vp1}:ssvp1]ip^s$	This study
SG200Δvp1-mpvp1	*a1 mfa2 bW2 Δvp1* $ip^r[P_{vp1}:mpvp1]ip^s$	This study
SG200Δvp1-SP_{vp1}-uhvp1	*a1 mfa2 bW2 Δvp1* $ip^r[P_{vp1}:SP_{vp1}:uhvp1]ip^s$	This study
SG200Δvp1-SP_{vp1}-srvp1	*a1 mfa2 bW2 Δvp1* $ip^r[P_{vp1}:SP_{vp1}:srvp1]ip^s$	This study
SG200Δvp1-otef-dSP-HA-vp1	*a1 mfa2 bW2 Δvp1* $ip^r[P_{otef}:dSP\text{-}HA\text{-}vp1]ip^s$	This study
SG200Δvp1-vp1(Δnls)	*a1 mfa2 bW2 Δvp1* $ip^r[P_{vp1}:vp1(\Delta nls)]ip^s$	This study
SG200Δvp1-otef-vp1-HA	*a1 mfa2 bW2 Δvp1* $ip^r[P_{otef}:vp1\text{-}HA]ip^s$	This study
SG200Δvp1-otef-vp1(Δnls)-HA	*a1 mfa2 bW2 Δvp1* $ip^r[P_{otef}:vp1(\Delta nls)\text{-}HA]ip^s$	This study
SG200-otef-SP-Cmu1-HA	*a1 mfa2 bW2* $ip^r[P_{otef}:SP\text{-}Cmu1\text{-}HA]ip^s$	Djamei et al., 2011
SG200Δvp1-otef-vp1-mCherryHA	*a1 mfa2 bW2 Δvp1* $ip^r[P_{otef}:vp1\text{-}mCherryHA]ip^s$	This study
SG200Δvp1-otef-vp1(nls*)-mCherryHA	*a1 mfa2 bW2 Δvp1* $ip^r[P_{otef}:vp1(nls^*)\text{-}mCherryHA]ip^s$	This study
SG200Δvp1-otef-vp1-mCherry	*a1 mfa2 bW2 Δvp1* $ip^r[P_{otef}:vp1\text{-}mCherry]ip^s$	This study
SG200-otef-mCherry-HA	*a1 mfa2 bW2* $ip^r[P_{otef}:mCherryHA]ip^s$	Provided by Regine Kahmann
SG200-P_{cmu1}-SP-mCherry-AvitagHA	*a1 mfa2 bW2* $ip^r[P_{cmu1}:SP\text{-}mCherry\text{-}AvitagHA]ip^s$	Provided by Regine Kahmann

Supplementary Reference

Djamei, A.; Schipper, K.; Rabe, F.; Ghosh, A.; Vincon, V.; Kahnt, J.; Osorio, S.; Tohge, T.; Fernie, A.R.; Feussner, I.; et al. Metabolic priming by a secreted fungal effector. *Nature* **2011**, *478*, 395-398, doi:10.1038/nature10454.

Dutheil, J.Y.; Mannhaupt, G.; Schweizer, G.; C, M.K.S.; Munsterkotter, M.; Guldener, U.; Schirawski, J.; Kahmann, R. A Tale of Genome Compartmentalization: The Evolution of Virulence Clusters in Smut Fungi. *Genome Biol Evol* **2016**, *8*, 681-704, doi:10.1093/gbe/evw026.

Kamper, J.; Kahmann, R.; Bolker, M.; Ma, L.J.; Brefort, T.; Saville, B.J.; Banuett, F.; Kronstad, J.W.; Gold, S.E.; Muller, O.; et al. Insights from the genome of the biotrophic fungal plant pathogen Ustilago maydis. *Nature* **2006**, *444*, 97-101, doi:10.1038/nature05248.

Kijpornyongpan, T.; Mondo, S.J.; Barry, K.; Sandor, L.; Lee, J.; Lipzen, A.; Pangilinan, J.; LaButti, K.; Hainaut, M.; Henrissat, B.; et al. Broad Genomic Sampling Reveals a Smut Pathogenic Ancestry of the Fungal Clade Ustilaginomycotina. *Mol Biol Evol* **2018**, *35*, 1840-1854, doi:10.1093/molbev/msy072.

Konishi, M.; Hatada, Y.; Horiuchi, J. Draft Genome Sequence of the Basidiomycetous Yeast-Like Fungus Pseudozyma hubeiensis SY62, Which Produces an Abundant Amount of the Biosurfactant Mannosylerythritol Lipids. *Genome Announc* **2013**, *1*, doi:10.1128/genomeA.00409-13.

Lanver, D. et al. The biotrophic development of *Ustilago maydis* studied by RNAseq analysis. *Plant Cell* **25**, doi: 10.1105/tpc.17.00764 (2018).

Laurie, J.D.; Ali, S.; Linning, R.; Mannhaupt, G.; Wong, P.; Guldener, U.; Munsterkotter, M.; Moore, R.; Kahmann, R.; Bakkeren, G.; et al. Genome comparison of barley and maize smut fungi reveals targeted loss of RNA silencing components and species-specific presence of transposable elements. *Plant Cell* **2012**, *24*, 1733-1745, doi:10.1105/tpc.112.097261.

Lorenz, S.; Guenther, M.; Grumaz, C.; Rupp, S.; Zibek, S.; Sohn, K. Genome Sequence of the Basidiomycetous Fungus Pseudozyma aphidis DSM70725, an Efficient Producer of Biosurfactant Mannosylerythritol Lipids. *Genome Announc* **2014**, *2*, doi:10.1128/genomeA.00053-14.

Morita, T.; Koike, H.; Koyama, Y.; Hagiwara, H.; Ito, E.; Fukuoka, T.; Imura, T.; Machida, M.; Kitamoto, D. Genome Sequence of the Basidiomycetous Yeast Pseudozyma antarctica T-34, a Producer of the Glycolipid Biosurfactants Mannosylerythritol Lipids. *Genome Announc* **2013**, *1*, e0006413, doi:10.1128/genomeA.00064-13

Müller, P.; Aichinger, C.; Feldbrügge, M.; Kahmann, R. The MAP kinase kpp2 regulates mating and pathogenic development in Ustilago maydis. *Mol Microbiol* **1999**, *34*, 1007-1017, doi:10.1046/j.1365-2958.1999.01661.x.

Rabe, F.; Bosch, J.; Stirnberg, A.; Guse, T.; Bauer, L.; Seitner, D.; Rabanal, F.A.; Czedik-Eysenberg, A.; Uhse, S.; Bindics, J.; et al. A complete toolset for the study of Ustilago bromivora and Brachypodium sp. as a fungal-temperate grass pathosystem. *Elife* **2016**, *5*, doi:10.7554/eLife.20522.

Redkar, A.; Hoser, R.; Schilling, L.; Zechmann, B.; Krzymowska, M.; Walbot, V.; Doehlemann, G. A Secreted Effector Protein of Ustilago maydis Guides Maize Leaf Cells to Form Tumors. *Plant Cell* **2015**, *27*, 1332-1351, doi:10.1105/tpc.114.131086.

Schirawski, J.; Mannhaupt, G.; Munch, K.; Brefort, T.; Schipper, K.; Doehlemann, G.; Di Stasio, M.; Rossel, N.; Mendoza-Mendoza, A.; Pester, D.; et al. Pathogenicity determinants in smut fungi revealed by genome comparison. *Science* **2010**, *330*, 1546-1548, doi:10.1126/science.1195330

Sharma, R.; Mishra, B.; Runge, F.; Thines, M. Gene loss rather than gene gain is associated with a host jump from monocots to dicots in the Smut Fungus Melanopsichium pennsylvanicum. *Genome Biol Evol* **2014**, *6*, 2034-2049, doi:10.1093/gbe/evu148.

Zambanini, T.; Buescher, J.M.; Meurer, G.; Wierckx, N.; Blank, L.M. Draft Genome Sequence of Ustilago trichophora RK089, a Promising Malic Acid Producer. *Genome Announc* **2016**, *4*, doi:10.1128/genomeA.00749-16.
